# Induction of BIM Is Essential for Apoptosis Triggered by EGFR Kinase Inhibitors in Mutant EGFR-Dependent Lung Adenocarcinomas

**DOI:** 10.1371/journal.pmed.0040294

**Published:** 2007-10-09

**Authors:** Yixuan Gong, Romel Somwar, Katerina Politi, Marissa Balak, Juliann Chmielecki, Xuejun Jiang, William Pao

**Affiliations:** 1 Pao Laboratory, Human Oncology and Pathogenesis Program, Memorial Sloan-Kettering Cancer Center, New York, New York, United States of America; 2 Varmus Laboratory, Cancer Biology and Genetics Program, Memorial Sloan-Kettering Cancer Center, New York, New York, United States of America; 3 Cell Biology Program, Memorial Sloan-Kettering Cancer Center, New York, New York, United States of America; University of California Los Angeles, United States of America

## Abstract

**Background:**

Mutations in the *epidermal growth factor receptor* (*EGFR*) gene are associated with increased sensitivity of lung cancers to kinase inhibitors like erlotinib. Mechanisms of cell death that occur after kinase inhibition in these oncogene-dependent tumors have not been well delineated. We sought to improve understanding of this process in order to provide insight into mechanisms of sensitivity and/or resistance to tyrosine kinase inhibitors and to uncover new targets for therapy.

**Methods and Findings:**

Using a panel of human lung cancer cell lines that harbor *EGFR* mutations and a variety of biochemical, molecular, and cellular techniques, we show that EGFR kinase inhibition in drug-sensitive cells provokes apoptosis via the intrinsic pathway of caspase activation. The process requires induction of the proapoptotic BH3-only BCL2 family member BIM (i.e., BCL2-like 11, or BCL2L11); erlotinib dramatically induces BIM levels in sensitive but not in resistant cell lines, and knockdown of BIM expression by RNA interference virtually eliminates drug-induced cell killing in vitro. BIM status is regulated at both transcriptional and posttranscriptional levels and is influenced by the extracellular signal-regulated kinase (ERK) signaling cascade downstream of EGFR. Consistent with these findings, lung tumors and xenografts from mice bearing mutant EGFR-dependent lung adenocarcinomas display increased concentrations of Bim after erlotinib treatment. Moreover, an inhibitor of antiapoptotic proteins, ABT-737, enhances erlotinib-induced cell death in vitro.

**Conclusions:**

In drug-sensitive *EGFR* mutant lung cancer cells, induction of BIM is essential for apoptosis triggered by EGFR kinase inhibitors. This finding implies that the intrinsic pathway of caspase activation may influence sensitivity and/or resistance of *EGFR* mutant lung tumor cells to EGFR kinase inhibition. Manipulation of the intrinsic pathway could be a therapeutic strategy to enhance further the clinical outcomes of patients with *EGFR* mutant lung tumors.

## Abstract 2

Using a panel of human drug-sensitive *EGFR* mutant lung cancer cells, William Pao and colleagues show that induction of BIM, a member of the BCL2 family, is essential for apoptosis triggered by EGFR kinase inhibitors.

## Editors' Summary

**Background.:**

Lung cancer, a common type of cancer, has a very low cure rate. Like all cancers, it occurs when cells begin to divide uncontrollably because of changes (mutations) in their genes. Chemotherapy drugs kill these rapidly dividing cells but, because some normal tissues are sensitive to these agents, it is hard to destroy the cancer without causing serious side effects. Recently, “targeted” therapies have brought new hope to some patients with cancer. These therapies attack the changes in cancer cells that allow them to divide uncontrollably but leave normal cells unscathed. One of the first molecules for which a targeted therapy was developed was the epidermal growth factor receptor (EGFR). In normal cells, messenger proteins bind to EGFR and activate its “tyrosine kinase,” an enzyme that sticks phosphate groups on tyrosine (an amino acid) in other proteins. These proteins then tell the cell to divide. Alterations to this signaling system drive uncontrolled cell division in some cancers so blocking the EGFR signaling pathway should stop these cancers growing. Indeed, some lung cancers with mutations in the tyrosine kinase of EGFR shrink dramatically when treated with gefitinib or erlotinib, two tyrosine kinase inhibitors (TKIs).

**Why Was This Study Done?:**

TKI-sensitive lung cancers shrink when treated with TKIs because of drug-induced cell death, but what are the molecular mechanisms underlying this death? A better understanding of how TKIs kill cancer cells might provide new insights into why not all cancer cells with mutations in *EGFR* (the gene from which EGFR is made) are sensitive to TKIs. It might also uncover new targets for therapy. TKIs do not completely kill lung cancers, but if the mechanism of TKI-induced cell death were understood, it might be possible to enhance their effects. In this study, the researchers have investigated how cell death occurs after kinase inhibition in a panel of human lung cancer cell lines (cells isolated from human tumors that grow indefinitely in dishes) that carry *EGFR* mutations.

**What Did the Researchers Do and Find?:**

The researchers show, first, that erlotinib induces a type of cell death called apoptosis in erlotinib-sensitive cell lines but not in resistant cell lines. Apoptosis can be activated by two major pathways. In this instance, the researchers report, the so-called “intrinsic” pathway activates apoptosis. This pathway is stimulated by proapoptotic members of the BCL2 family of proteins and is blocked by antiapoptotic members, so the researchers examined the effect of erlotinib treatment on the expression of BCL2 family members in the *EGFR* mutant cell lines. Erlotinib treatment increased the expression of the proapoptotic protein BIM in sensitive but not in resistant cell lines. It also removed phosphate groups from BIM—dephosphorylated BIM is a more potent proapoptotic protein. Conversely, blocking BIM expression using a technique called RNA interference virtually eliminated the ability of erlotinib to kill *EGFR* mutant cell lines. The researchers also report that erlotinib treatment increased BIM expression in erlotinib-sensitive lung tumors growing in mice and that an inhibitor of the anti-apoptotic protein BCL2 enhanced erlotinib-induced death in drug-sensitive cells growing in dishes.

**What Do These Findings Mean?:**

These findings indicate that BIM activity is essential for the apoptosis triggered by TKIs in drug-sensitive lung cancer cells that carry *EGFR* mutations, and that treatment of these cells with TKIs induces both the expression and dephosphorylation of BIM. The finding that the intrinsic pathway of apoptosis activation is involved in TKI-induced cell death suggests that changes in this pathway (possibly mutations in some of its components) might influence the sensitivity of *EGFR* mutant lung cancers to TKIs. Finally, these findings suggest that giving drugs that affect the intrinsic pathway of apoptosis activation at the same time as TKIs might further improve the clinical outcome for patients with *EGFR* mutant tumors. Such combinations will have to be tested in clinical trials before being used routinely.

**Additional Information.:**

Please access these Web sites via the online version of this summary at http://dx.doi.org/10.1371/journal.pmed.0040294.

- US National Cancer Institute information for patients and professionals on lung cancer (in English and Spanish)
- Information for patients from Cancer Research UK on lung cancer including information on treatment with TKIs
- Wikipedia pages on apoptosis, epidermal growth factor receptor, and BCL-2 proteins (note that Wikipedia is a free online encyclopedia that anyone can edit; available in several languages)
- Information for patients from Cancerbackup on erlotinib and gefitinib

## Introduction

Lung adenocarcinomas that harbor somatic kinase domain mutations in the epidermal growth factor receptor (EGFR) are highly likely to respond to the EGFR tyrosine kinase inhibitors gefitinib (Iressa) and erlotinib (Tarceva) [1–3]. One possible explanation for this phenomenon is that the cancer cells are “addicted” to signaling via the mutant EGFRs and die when the mutant oncoprotein is inactivated. However, specific mechanisms underlying erlotinib-induced cell death have not been well delineated.

To date, drug responses have been characterized in limited detail in a few drug-sensitive human lung adenocarcinoma cell lines that harbor *EGFR* mutations. These studies indicate that EGFR kinase inhibitors can induce the activation of cysteine aspartyl-specific proteases (caspases) in *EGFR* mutant cells [4–7]. Similarly, analyses of mouse models of drug-sensitive mutant EGFR-driven lung adenocarcinomas have demonstrated that erlotinib induces caspase activation [8,9]. However, none of these studies addressed the molecular mechanisms by which caspases are activated.

Two major pathways of caspase activation exist: the extrinsic pathway, triggered by “death receptor”-ligand binding (such as FASL to FAS), and the intrinsic pathway, triggered by cellular stress, such as occurs after cytokine deprivation, DNA damage, and anoikis (reviewed in [10]). BCL2 family proteins are a critical feature of the intrinsic pathway. Overexpression of some antiapoptotic family members, such as BCL2 and BCL-xL (also called BCL2-like ligand 1 [BCL2L1]), can prevent apoptosis induced by withdrawal of growth factor [11,12]. Control of stability of the BCL2-related antiapoptotic myeloid cell leukemia sequence 1 (MCL1) protein is also an important mechanism for the regulation of apoptosis by growth factors [13]. BCL2-associated X protein (BAX) and BCL2 antagonist/killer (BAK), on the other hand, are proapoptotic BCL2 family members. Deletion of both renders cells highly resistant to apoptosis following growth factor withdrawal [14,15]. During apoptosis, BAX translocates to mitochondria, causing release of cytochrome *c* into the cytosol [16].

A third proapoptotic subgroup of the BCL2 family includes BIM (also known as BCL2L11 [BCL2-like 11]), BAD (BCL2-antagonist of cell death), and BBC3 (BCL2-binding component 3; also called PUMA), which all contain a “BH3” protein domain. These BH3-only members of the BCL2 family promote apoptosis when overexpressed [17,18] by inhibiting antiapoptotic members and/or directly activating BAX/BAK [19,20]. Bim is induced in interleukin 3 (IL3)-dependent murine hematopoietic cells by IL-3 deprivation but not by other apoptotic triggers, such as DNA damage or Fas [21]. BIM is one of the most potent proapoptotic BH3-only proteins; unlike BAD, BIM binds to all prosurvival BCL2 family members with high affinity [19]. Recent analyses of IL3-dependent cell lines from mice lacking the genes for Bad, Bim, Puma, Bad and Bim, or Bax and Bak, showed that while Bad and Bim were not required for cell death following IL3 withdrawal, cell death did in part depend upon Puma [22]. Mechanisms of death in leukemic cells that depend upon signaling from the Bcr-Abl oncoprotein for survival may be different from those in cells that depend upon IL3, as studies using RNA interference and cells from gene-targeted mice revealed that the combined loss of Bim and Bad abrogates cell killing induced by the kinase inhibitor imatinib (Gleevec) [23].

In this study, we sought to determine the mode of cell death induced by EGFR kinase inhibitors in drug-sensitive human lung adenocarcinoma cells that harbor *EGFR* mutations. We hoped that an improved understanding of this process could provide insight into mechanisms of sensitivity and/or resistance to tyrosine kinase inhibitors (TKIs) and uncover new targets for therapy.

## Methods

### Cell Culture

The human lung adenocarcinoma cell lines H3255, PC-9, H1650, and H1975 were described previously [4–7,24]. Cells were maintained in a humidified incubator with 5% CO_2_ at 37 °C in RPMI 1640 medium (ATCC, Manassas, VA) supplemented with 10% fetal bovine serum and pen-strep solution (both from Gemini Bio-Products, West Sacramento, CA; final concentrations of 100 U/ml penicillin G and 100 μg/ml streptomycin, respectively). NIH 3T3 cells stably transfected with various *EGFR* cDNAs were kindly provided by M. Meyerson [25]. BCL-xL-overexpressing PC-9 cells were generated by transfecting parental cells with pcDNA3.1(−) hygromycin vector carrying a *BCL-xL* cDNA [26] using Fugene 6 transfection reagent (Roche Diagnostics, Indianapolis, IN). Resistant clones were selected in 250 μg/ml hygromycin B (Invitrogen, Carlsbad, CA) for 12 d, isolated using cloning cylinders, and subsequently expanded in hygromycin B-containing medium. Control cells were transfected with empty pcDNA3.1(−) hygromycin vector.

### Reagents

Erlotinib was synthesized by the Organic Synthesis Core Facility at Memorial Sloan-Kettering Cancer Center. Gefitinib was kindly provided by AstraZeneca (Wilmington, DE). ABT-737 and its inactive enantiomer were kindly provided by Abbott Laboratories (Abbott Park, IL). The Vybrant Apoptosis Assay Kit #2 was from Invitrogen. CellTiter Blue Reagent and APO-ONE Homogenous Caspase3/7 assay kits were from Promega (Madison, WI). Propidium iodide and actinomycin D were from Sigma-Aldrich (St. Louis, MO). CL-387,785, U0126, PD98059, LY294002, and wortmannin were from Calbiochem (San Diego, CA).

### Antibodies

Cytochrome *c*-, MCL1-, and EGFR-specific antibodies were from BD Biosciences (San Jose, CA). Anti-actin antibody was from Sigma-Aldrich. Anti-BCL-2, -BCL-xL, -BAX, -BAD, -BIM, -PUMA, -pERK (Thr202/Tyr204), -ERK, -pAKT (Ser473), -AKT, and -pEGFR (Tyr1092) antibodies, and secondary antibodies including HRP-conjugated anti-mouse and anti-rabbit antibodies, were purchased from Cell Signaling Technology (Danvers, MA). (Note that EGFR has two numbering systems. The first denotes the initiating methionine in the signal sequence as amino acid −24. The second denotes the methionine as amino acid +1. Commercial antibodies, such as the Y1068-specific anti-phospho-EGFR, use the first nomenclature. In the second nomenclature, which we use here, Y1068 is Y1092.) BAK-specific antibody was from Lab Vision Corporation (Fremont, CA). L858R EGFR antisera was previously described [9]. Anti-total EGFR and FITC- and rhodamine-conjugated secondary antibodies were from Santa Cruz Biotechnology (Santa Cruz, CA).

### Cell Viability Assay

Cells were seeded in 96-well plates at a density of 5,000 cells in triplicates and treated with different concentrations of erlotinib on the following day. The viability of erlotinib-treated cells was measured at 24, 48, and 72 h post-treatment using CellTiter Blue Reagent (Promega). Caspase 3/7 activity was monitored in the same wells using the APO-ONE Homogenous Caspase 3/7 assay kit (Promega).

### Flow Cytometry Analysis

Cells were collected, washed with PBS, and fixed in 70% ethanol for at least 1 h. Prior to FACS analysis, cells were washed twice with cold PBS and resuspended in PBS containing 200 μg/ml RNase, 50 μg/ml propidium iodide (PI), and 0.1% sodium citrate. Flow cytometry was performed on a Becton-Dickinson FACSCalibur flow cytometer. Data were processed using FlowJo flow cytometry analysis software (http://www.flowjo.com/). Annexin V/PI apoptosis assays (Invitrogen) were performed according to manufacturer's instructions.

### Subcellular Fractionation

Subcellular fractionation was performed as described previously [26].

### Immunoblotting

Cells were scraped from 10 cm petri dishes, washed twice with PBS, and then incubated in lysis buffer [27] containing protease inhibitor cocktail (Roche Diagnostics), 40 mM sodium fluoride, and 1 mM sodium orthovanadate for 30 min. The supernatants were subjected to SDS-PAGE (gels from Invitrogen) followed by blotting with indicated antibodies and detected by Supersignal West Pico Luminol/Enhancer Solution (Pierce Biotechnology, Rockford, IL).

### Immunofluorescence and Confocal Imaging

Cells were grown in Lab-Tek II chambers (Nalge Nunc International, Naperville, IL), fixed in 4% (vol/vol) paraformaldehyde solution for 10 min at room temperature, permeabilized in 0.5% Triton X-100 solution for 10 min, incubated in blocking solution (5% BSA) for 1 h at room temperature and incubated with primary antibody (1:100) in 5% BSA overnight at 4 °C. After 1 h incubation with FITC- or rhodamine-conjugated secondary antibody (1:500), slides were mounted in Vectashield mounting medium (Vector Laboratories, Burlingame, CA). Digital images were obtained with a Leica inverted confocal microscope.

### Gene Silencing by siRNA

Cells were seeded into six-well plates at a density of 1.25 × 10^5^ cells/well. After 24 h, cells were transfected with various small interfering RNAs (siRNAs) against *GFP*, *BIM*, or *BAD,* using DharmaFECT 1 transfection reagent as per manufacturer's instructions. *GFP* siRNA and *BIM* siRNA-2 were obtained from the High Throughput Core Facility (Memorial Sloan-Kettering Cancer Center, New York, NY). *BAD* siRNA and *BIM* siRNA-1 were from Dharmacon (siGENOME SMARTpool, Dharmacon, Chicago, IL). Cells were harvested 48 h after transfection, and lysates were analyzed for BIM expression by immunoblotting analysis.

### Animal Husbandry and Genotyping

The generation, maintenance, genotyping, and drug treatment of bitransgenic lung tumor-bearing mice that express a tetracycline-inducible exon 19 deletion mutant (*EGFR* ^ΔL747–S752^) or the L858R mutant (*EGFR* ^L858R^) in mouse lung epithelia have been previously described [9]. Animals were humanely killed with a lethal dose of CO_2_ per institutional guidelines. The lungs were excised, flash-frozen in liquid nitrogen, and crushed for protein extraction in lysis buffer supplemented with protease and phosphatase inhibitors.

For xenograft studies, the hind flanks of 4- to 6-wk-old female athymic (*nu/nu*) nude mice (NCI-Frederick Cancer Center, Frederick, MD) were injected subcutaneously with 10 × 10^6^ PC-9 cells resuspended in 100 μl of Matrigel (BD Biosciences). Tumor-bearing animals (12 d after injection) were administered either vehicle control or 25 mg/kg erlotinib intraperitoneally every 12 h × 2 doses and then humanely killed 24 h after the first injection. Xenografts were excised, flash-frozen in liquid nitrogen, and crushed for protein extraction in lysis buffer supplemented with protease and phosphatase inhibitors.

All animals were kept in specific pathogen-free housing with abundant food and water under guidelines approved by the Memorial Sloan-Kettering Cancer Center Institutional Animal Care and Use Committee and Research Animal Resource Center.

## Results

### EGFR Kinase Inhibition Induces G1 Cell Cycle Arrest in Drug-Sensitive Lung Adenocarcinoma Cells with *EGFR* Mutations

A number of lung adenocarcinoma cell lines have been reported to harbor drug-sensitizing *EGFR* mutations [4–7,24]. To verify these findings, we obtained four of these cell lines and determined their mutation status by direct Sanger-based resequencing of *EGFR* exons 18–24 (Table 1). As reported, PC-9 and H1650 cells contained the in-frame exon 19 deletion that eliminates amino acids ELREA (del E746–A750). H3255 and H1975 harbored the exon 21 T→G point mutation at nucleotide position 2,573, which substitutes arginine for leucine at position 858 (L858R). H1975 cells also contained the exon 20 C→T mutation at nucleotide position 2,369. This change leads to substitution of methionine for threonine at position 790 (T790M) and is associated with acquired resistance to gefitinib and erlotinib [24].

**Table 1:**
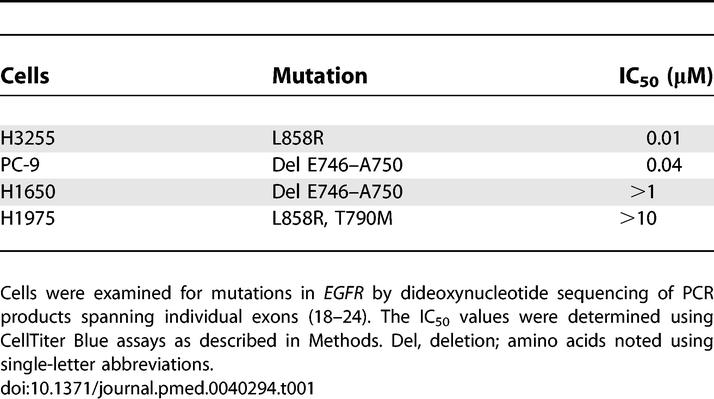
Sensitivity of *EGFR-*Mutant Lung Adenocarcinoma Cell Lines to Erlotinib

**Figure 1:**
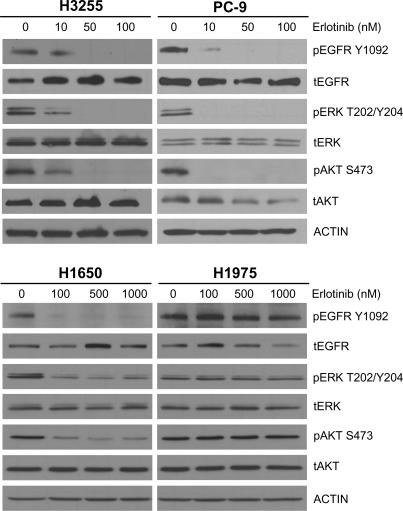
Effects of Erlotinib on EGFR Signaling Pathways in H3255, PC-9, H1650, and H1975 Cells Cells were treated with the indicated concentrations of erlotinib for 6 h. Cell lysates were analyzed by immunoblotting, using the indicated antibodies. t, total protein; p, phospho-protein.

We established the sensitivity of these cell lines to erlotinib using a cell viability assay that measures a colorimetric signal produced by conversion of resazurin to resorufin, which is directly proportional to the numbers of viable cells. Cells were grown in 10% serum for 72 h in different concentrations of erlotinib. As previously reported, the concentration of drug required for 50% growth inhibition (IC_50_) of two lines—H3255 and PC-9—was in the nanomolar range (10 nM and 40 nM respectively) (Table 1). The IC_50_ values corresponded with the degrees of inhibition of phosphorylation of EGFR and key components of downstream signaling cascades (i.e., ERK and AKT), as seen by immunoblotting lysates derived from the lines treated with various concentrations of drug with phospho-specific antibodies (Figure 1). H1650 cells had an intermediate sensitivity (Table 1). Consistent with this finding, H1650 lysates displayed incomplete inhibition of ERK and AKT pathways (Figure 1), the latter possibly due to lack of expression of the phosphatase and tensin homolog (PTEN) protein, which negatively regulates the phosphatidylinositol 3-kinase signaling cascade downstream of EGFR [28]. H1975 cells were insensitive to erlotinib due to the drug-resistant T790M mutation [24].

**Figure 2:**
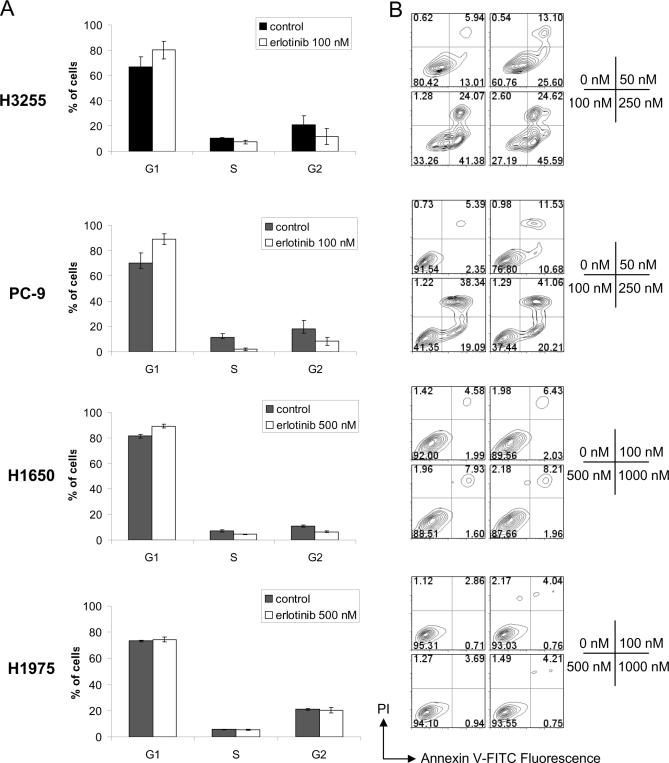
Erlotinib Induces G1 Cell Cycle Arrest and Apoptosis in Drug-Sensitive Lung Adenocarcinoma Cell Lines That Harbor *EGFR* Mutations (A) Cell lines treated with either DMSO or indicated concentrations of erlotinib for 24 h were used for cell cycle analysis as described in the Methods. Data represent the mean ± standard deviation of three independent experiments. (B) Cell lines treated with either DMSO or different concentrations of erlotinib for 48 h were used for annexin V/PI apoptosis assays as described in Methods.

Sensitivity of cells to erlotinib could result from an arrest of cells in a specific stage of the cell cycle. To investigate this possibility, we treated the four cell lines with either DMSO or erlotinib and then analyzed their DNA content by FACS after staining with the DNA-intercalating dye PI. After 24 h of 100 nM erlotinib treatment, both drug-sensitive cell lines (H3255 and PC-9) showed an increase in the proportion of cells in the G1 phase and a decrease in the proportion of cells in S phase, compared to untreated cells (Figure 2A). These results indicate that erlotinib induces G1 cell cycle arrest in drug-sensitive lung adenocarcinoma cells with *EGFR* mutations. H1650 cells, treated with a higher dose (500 nM) of erlotinib, displayed only a slight increase in the proportion of cells in the G1 phase compared to control-treated cells. Minimal changes were observed after treatment of drug-resistant H1975 cells.

### EGFR Kinase Inhibition Induces Apoptosis in H3255 and PC-9 Cells

We next investigated whether erlotinib also induced apoptosis in the cell lines with nanomolar sensitivity. Cells treated with DMSO or different concentrations of erlotinib for 48 h were stained with FITC-conjugated annexin V and PI, and percentages of apoptotic cells were measured by FACS. (The phospholipid-binding protein annexin V has a high affinity for phosphatidylserine, which is translocated from the inner to the outer leaflet of the plasma membrane during the early phases of apoptosis, and PI penetrates the plasma membrane of cells only when membrane integrity is breached, as occurs in the later stages of apoptosis.) After exposure to escalating concentrations of drug, PC-9 and H3255 cells demonstrated dose-dependent increases in the percentages of annexin V- and PI-positive cells (Figure 2B). Consistent with these findings, the activity of downstream “executioner” caspases, caspases 3 and 7, in lysates from cells treated with erlotinib at various concentrations for various times, was also increased in both dose- and time-dependent fashion (Figure S1). By contrast, minimal increases in the percentage of annexin V- and PI-positive cells were observed in drug-treated H1650 and H1975 cells, even at higher doses of erlotinib (1 μM) (Figure 2B).

### Mutant EGFR-Dependent Cells Die via the Intrinsic Pathway of Apoptosis

We subsequently examined mechanisms of apoptosis in the two cell lines that undergo apoptosis in response to erlotinib. Caspases can be activated via two major pathways: the extrinsic pathway, triggered by the binding of ligands to “death receptors,” and the intrinsic pathway, triggered by cellular stress, such as occurs after cytokine deprivation, DNA damage, and anoikis [10]. Since inhibition of EGFR kinase activity by erlotinib is similar to growth factor withdrawal, we investigated whether erlotinib induced the intrinsic pathway of caspase activation in PC-9 and H3255 cells by a stress-related mechanism.

**Figure 3:**
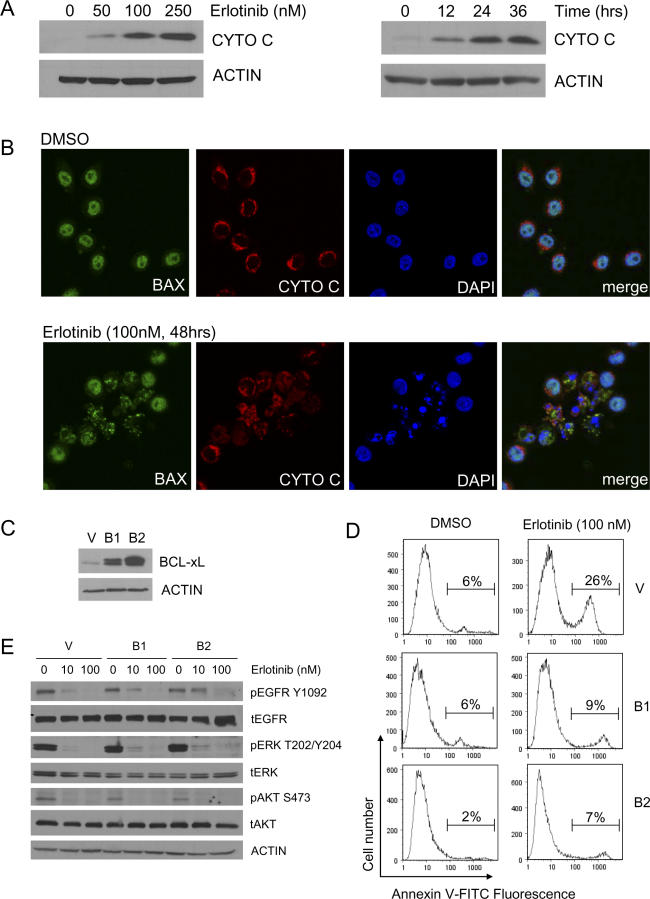
Erlotinib-Induced Cell Death Is Mediated by the Intrinsic Pathway of Apoptosis (A) Erlotinib induces time- and dose-dependent cytochrome *c* release in H3255 cells. H3255 cells were treated with different concentrations of drug for 24 h, or 100 nM erlotinib for the indicated times. Cytosolic fractions were obtained through differential centrifugation as described in the Methods and used for immunoblotting. (B) Erlotinib induces colocalization of BAX and cytochrome *c* in PC-9 cells undergoing apoptosis. Cells were grown in Lab-Tek II chambers and treated with 100 nM erlotinib or DMSO for 48 h, fixed and immunostained with antibodies against BAX (green) and cytochrome *c* (red) or with DAPI to detect DNA (blue). Cells were imaged by confocal microscopy. (C) PC-9 cells were stably transfected with cDNAs encoding BCL-xL. Two stable clones (B1 and B2) showed elevated levels of BCL-xL compared to cells transfected with expression vector alone, as shown by immunoblotting. (D) BCL-xL overexpression blocks erlotinib-induced apoptosis in PC-9 cells. Stable clones expressing empty vector (V) or BCL-xL (B1 and B2) were treated with DMSO or 100 nM erlotinib for 48 h and percentages of apoptotic cells were estimated using annexin V/PI apoptosis assays. (E) BCL-xL overexpression does not affect the ability of erlotinib to inhibit EGFR signaling pathways in PC-9 cells. Stable clones were treated with 100 nM erlotinib for 6 h. The phosphorylation status of proteins involved in EGFR signaling was examined by immunoblotting. t, total protein; p, phospho-protein.

One hallmark of the intrinsic pathway of apoptosis is cytochrome *c* release from mitochondria [29]. We found by immunoblotting cytosolic fractions from H3255 cells treated with erlotinib that cytochrome *c* levels increased within the cytosol in both a time- and concentration-dependent manner (Figure 3A). Similar results were obtained with cytosolic fractions from drug-treated PC-9 cells (unpublished data).

Changes in the localization of proapoptotic BAX are another hallmark of the intrinsic pathway of apoptosis [29]. Immunostaining followed by confocal microscopy revealed that at baseline, BAX was found in the nuclei of both PC-9 and H3255 cells (Figure 3B and unpublished data); the nuclear localization of BAX in human lung cancer cell lines has been previously reported [30,31]. Following erlotinib treatment, BAX was found in the cytoplasm in apoptotic cells. The distribution of cytochrome *c* changed from punctate to more diffuse, consistent with the release of cytochrome *c* from mitochondria. Merging of the two fluorescent signals revealed some overlap between BAX and cytochrome *c* staining. These observations suggest that BAX translocates to mitochondria during erlotinib-induced apoptosis in mutant EGFR-dependent lung cancer cells.

To verify further whether erlotinib-induced apoptosis is mediated through the intrinsic apoptotic pathway, we examined effects of erlotinib on PC-9 cells that stably overexpress the antiapoptotic BCL-2 family member BCL-xL. Two stable clones (B1 and B2) were derived, and both displayed significantly higher levels of BCL-xL than did cells transfected with vector alone (Figure 3C). Compared to vector-transfected cells, in which 100 nM erlotinib increased the fraction of annexin V-positive cells by 20% over DMSO-treated cells, drug treatment in cells overexpressing BCL-xL increased the fraction of apoptotic cells by only 3%–5% over DMSO-treated controls (Figure 3D). As expected, BCL-xL did not affect the inhibition of EGFR signaling by erlotinib. The drug still inhibited the autophosphorylation of EGFR as well as the phosphorylation of ERK and AKT (Figure 3E). These results collectively indicate that erlotinib-induced apoptosis in mutant EGFR-dependent lung adenocarcinoma cells is mediated predominantly by the intrinsic apoptotic pathway.

### The Status of BIM Is Dramatically Altered by Inhibition of EGFR Kinase Activity in Cells Dependent upon Mutant EGFR Signaling for Survival

**Figure 4:**
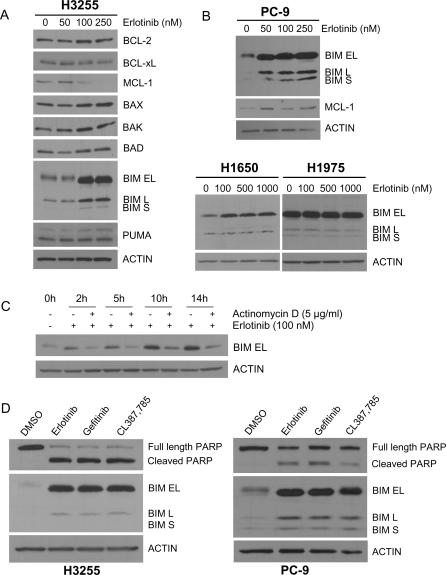
BIM Status Is Dramatically Altered by Inhibition of EGFR Kinase Activity in Cells Dependent upon Mutant EGFR Signaling for Survival (A) Effects of erlotinib treatment on proapoptotic and antiapoptotic proteins in H3255 cells. H3255 cells were treated with different concentrations of erlotinib for 24 h. Cell lysates were examined by immunoblotting using antibodies specific for the indicated proteins. (B) Erlotinib induces dephosphorylation and increased levels of BIM in drug-sensitive PC-9 cells but not in drug-resistant H1650 or H1975 cells. Cells were treated with different concentrations of erlotinib for 24 h. Cell lysates were analyzed by immunoblotting with antibodies that recognize the indicated proteins. (C) Increased gene transcription contributes to the induction of BIM protein expression after erlotinib treatment. H3255 cells were treated with erlotinib (100 nM) in the presence or absence of actinomycin D (5 μg/ml) for the indicated times. Cell lysates were analyzed by immunoblotting using the indicated antibodies. (D) Multiple EGFR kinase inhibitors affect BIM status. H3255 (left blot) and PC-9 (right blot) cells were treated with erlotinib (100 nM), gefitinib (1 μM), or CL-387,785 (100 nM) for 24 h and 48 h, respectively. Cell lysates were analyzed by immunoblotting with antibodies that recognize the indicated proteins.

The intrinsic pathway of apoptosis is regulated by BCL2 family member proteins, some of which are antiapoptotic (e.g., BCL2, BCL-xL, MCL1) and some proapoptotic (e.g., the BAX family members BAX and BAK, and the BH3-only proteins BIM, BAD, and PUMA) [17,18]. To determine which BCL2 family members are potentially involved in the maintenance of tumors by mutant EGFR, we studied the effect of erlotinib treatment on the expression of BCL2, BCL-xL, MCL1, BAX, BAK, BIM, BAD, and PUMA in H3255 cells. We found that the expression levels of only the antiapoptotic protein MCL1 and the proapoptotic BH3-only protein BIM were significantly altered by the drug (Figure 4A).

MCL1 protein is rapidly degraded by the proteasome and its elimination is required for the initiation of apoptosis following ultraviolet radiation in HeLa cells [32]. To investigate if down-regulation of MCL1 is essential for erlotinib-induced apoptosis in lung adenocarcinoma cells, we blocked MCL1 degradation with the proteasome inhibitor MG132 in H3255 cells. Despite the efficient inhibition of MCL1 degradation by MG132, erlotinib was still able to induce apoptosis as measured by an increase in PARP (poly ADP ribose polymerase) cleavage (Figure S2). Moreover, MCL1 levels remained constant in erlotinib-treated PC-9 cells, which also undergo apoptosis in response to drug treatment (Figure 4B). These results collectively indicate that MCL1 elimination is not essential for erlotinib-induced apoptosis in mutant EGFR-dependent cells.

We next focused our attention on BIM. Immunoblotting analysis of the four cell lines revealed that the levels of all three major splicing isoforms—BIM-extra long (EL), BIM-long (L), and BIM-short (S)—were induced after erlotinib treatment in drug-sensitive H3255 and PC-9 cells but not in drug-resistant H1650 and H1975 cells (Figure 4A and 4B). Moreover, in the drug-sensitive but not in the drug-resistant lines, erlotinib appeared to induce significant dephosphorylation of BIM EL, as this isoform migrated faster in polyacrylamide gel electrophoresis after drug treatment (Figure 4A and 4B). This finding is notable because others have shown that dephosphorylation of BIM results in an increase in its proapoptotic function [33,34].

*BIM* has been shown to be regulated transcriptionally [35]. To investigate whether the induction of BIM protein by erlotinib occurred at the transcriptional level in H3255 cells, we measured BIM proteins in lysates from cells treated for varying amounts of time with either erlotinib alone or with erlotinib and the RNA polymerase II inhibitor, actinomycin D, which inhibits mRNA transcription (Figure 4C). BIM EL levels rose within 2 h after treatment with the kinase inhibitor alone and continued to increase up to at least 14 h. Cotreatment with actinomycin D efficiently blocked most of this increase. Consistent with this, transcriptional profiling of H3255 cells treated with erlotinib showed that levels of *BIM* mRNA increased significantly 2.5-fold by 12 h after treatment (unpublished data). These data suggest that augmented transcription of *BIM* can account for at least part of the increase in BIM protein after treatment of mutant EGFR-dependent cells with erlotinib.

Finally, to determine if induction of BIM was unique to erlotinib, we examined the status of BIM in H3255 and PC-9 cells treated with erlotinib (100 nM) or two other EGFR inhibitors: the related reversible EGFR inhibitor gefitinib (1 μM) and the irreversible EGFR inhibitor CL-387,785 (100 nM) [36]. All three drugs induced apoptosis in these cell lines, as indicated by the presence of PARP cleavage products in cell lysates (Figure 4D). Consistent with these findings, all three EGFR inhibitors induced the expression and dephosphorylation of BIM EL. These data indicate that the induction of BIM is triggered by various EGFR inhibitors and is not a specific effect of erlotinib.

### BIM Is Required for Erlotinib-Induced Apoptosis

**Figure 5:**
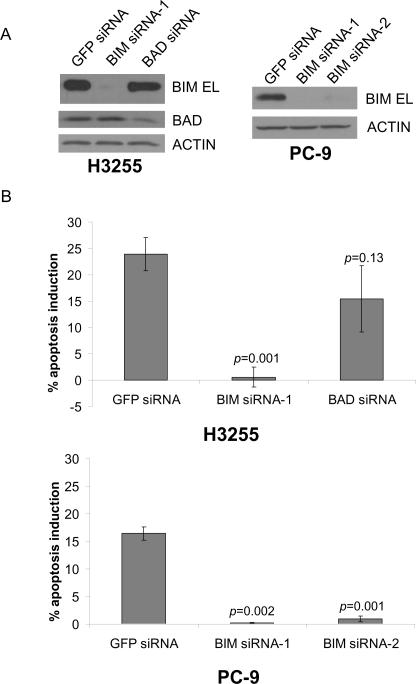
The BH3-Only Protein BIM Is Required for Erlotinib-Induced Apoptosis (A) Knockdown of BIM expression by siRNA transfection. H3255 and PC-9 cells were transiently transfected with various siRNAs against *GFP*, *BIM*, or *BAD*. Two different siRNAs against *BIM* were used in PC-9 cells. Cells were harvested 48 h after transfection and lysates were examined for BIM expression by immunoblotting. (B) Knockdown of BIM expression completely blocks erlotinib-induced apoptosis. At 48 h post siRNA transfection, H3255 cells were treated with DMSO or 100 nM erlotinib for an additional 24 h. At 24 h after siRNA transfection, PC-9 cells were treated with DMSO or 100 nM erlotinib for an additional 48 h. Cells were harvested, and the percentage of apoptotic cells was determined by annexin V/PI apoptosis assays. Annexin V-positive cells were calculated as apoptotic cells. The average percent ± standard deviation of apoptotic cells induced by erlotinib in cells transfected with *GFP*, *BIM*, or *BAD* siRNA was derived from three independent experiments. The indicated *p*-values were calculated using a Welch two-sample t-test.

To examine the role of BIM in erlotinib-induced cell killing in more detail, we used siRNAs to suppress BIM expression almost completely in both H3255 and PC-9 cells (Figure 5A). Two independent siRNAs were utilized to knock down BIM in PC-9 cells.

Knockdown effectively abolished erlotinib-induced killing in both cell lines (Figure 5B). By comparison, cells transfected with control siRNA against *GFP* continued to undergo apoptosis in response to erlotinib. In each of three independent experiments, the difference in percent of apoptotic cells induced by erlotinib in H3255 and PC-9 cells was statistically significant (*p* = 0.001 for H3255 with *BIM* siRNA1, and *p* = 0.002 and 0.001 for PC-9 cells with *BIM* siRNA-1 and − 2, respectively; Welch two-sample t-test). As another comparison, we also examined the effect of knockdown of a different proapoptotic protein, BAD, on erlotinib-induced apoptosis in H3255 cells. Here, no statistically significant difference in rates of apoptosis was observed (*p* = 0.13; Welch two-sample t-test) (Figure 5B). However, despite multiple attempts with different siRNAs, we were unable to abolish BAD expression completely (unpublished data). Thus, we could not entirely exclude the possibility that BAD is also required for erlotinib-induced cell death.

### EGFR Signaling Influences BIM Expression and Phosphorylation Status Mainly via the ERK Pathway

**Figure 6:**
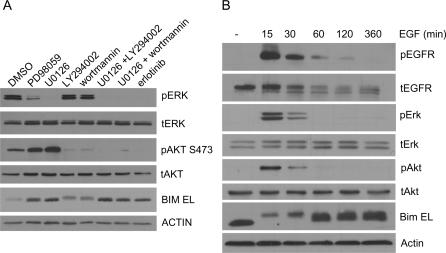
The Status of BIM Is Influenced by EGFR Signaling (A) BIM phosphorylation and protein levels are influenced by the ERK signaling pathway. H3255 cells were treated with a MEK inhibitor (PD98059, 50 μM; U0126, 10 μM), a PI3K inhibitor (LY294002, 50 μM; wortmannin, 100 nM every 2 h), both, or 100 nM erlotinib for 8 h. Cell lysates were analyzed by immunoblotting using the indicated antibodies. (B) NIH 3T3 cells transfected with wild-type *EGFR* cDNAs were serum-starved overnight and then stimulated with 100 ng/ml human EGF. After the indicated times, cells were harvested. Lysates were analyzed by immunoblotting, using the indicated antibodies. t, total protein; p, phospho-protein.

Mutant EGFRs activate both the ERK and phosphatidylinositol 3-kinase/protein kinase B (PI3K/AKT) signaling pathways [4,6]. Both cascades have been shown to regulate BIM phosphorylation, degradation, and activity, although in different cellular and biological contexts [33,34,37]. Since EGFR kinase inhibition by erlotinib diminishes activation of both ERK and AKT pathways in drug-sensitive cells but not in drug-resistant cells (Figures 1 and 6A), we next asked whether inhibition of both pathways is required for the increased expression and dephosphorylation of BIM. Thus, we examined the status of phospho-ERK, -AKT, and BIM in lysates from H3255 cells treated with erlotinib and compared results to those obtained from cells exposed to a mitogen-activated protein kinase kinase (MAP2K or MEK) inhibitor (PD98059 or U0126, which affect the ERK pathway), a PI3K inhibitor (wortmannin or LY294002), or both. We focused our attention primarily on the BIM EL isoform, because it is the major isoform detected in these cells, and the BIM EL isoform, but not BIM L and S, contains phosphorylation sites for ERK and AKT [33,34].

Treatment of H3255 cells with either MEK inhibitor led to an increase in BIM expression levels as well as the dephosphorylation of BIM (as indicated by the faster migrating form) (Figure 6A)—changes similar to that seen in erlotinib-treated cells. Treatment with the PI3K inhibitors alone had minimal effects on either BIM expression levels or phosphorylation status. Lysates from cells treated with the combination of MEK (U0126, Figure 6A) and PI3K inhibitors (wortmannin or LY294002, Figure 6A) did not show any additive effects on the induction of BIM EL, when compared with cells treated with the MEK inhibitor treatment alone. These data suggest that mutant EGFRs influence BIM expression and phosphorylation status mainly through the ERK signaling pathway. More precise mechanisms of BIM regulation remain to be elucidated.

Finally, to begin to determine how EGFR signaling affects BIM in an isolated cell system, we examined the effect of EGF stimulation on Bim in NIH 3T3 cells stably transfected with cDNAs encoding wild-type *EGFR* [25]. These mouse fibroblasts express very low levels of endogenous Egfr and its heterodimeric Erbb family members (i.e., Erbb2, Erbb3, and Erbb4). As expected, addition of exogenous EGF to serum-starved cells induced a rapid and massive phosphorylation of the receptor within 15 min, followed by a time-dependent decay in receptor activity (Figure 6B). Levels of total EGFR decreased after EGF stimulation, presumably due to internalization and lysosomal degradation of the receptor. These changes were accompanied by transient increases in both Erk and Akt signaling. In parallel with these changes, EGF stimulation induced a rapid phosphorylation of Bim as well as a decrease in Bim protein levels; others have shown that these changes correlate with decreased Bim activity [34,38]. BIM levels were restored quickly as levels of phospho-EGFR diminished. Thus, Bim protein levels and phosphorylation are tightly regulated by the EGFR signaling pathway. Moreover, EGFR stimulation by ligand can lead to changes suggestive of decreased BIM activity.

### Bim Expression and Phospho-EGF Receptor Status Are Inversely Correlated in Mouse Models

**Figure 7:**
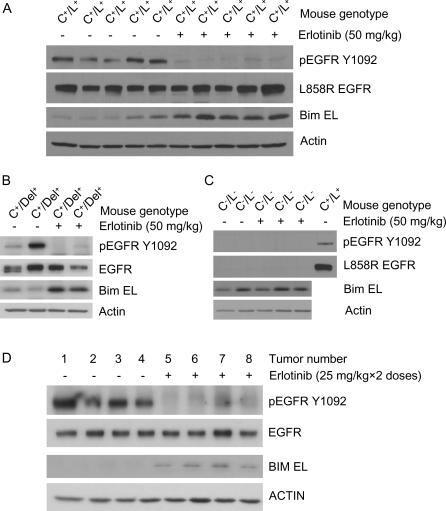
In Vivo Correlation of BIM/Bim Expression and Mutant EGFR Activity (A and B) Lung tumor-bearing mice that express a tetracycline-inducible L858R mutant (*EGFR* ^L858R^) (A) or an exon 19 deletion mutant (*EGFR* ^ΔL747–S752^) (B) were treated with a single dose of either vehicle control or 50 mg/kg erlotinib intraperitoneally. After 13 h, animals were sacrificed and lungs were harvested. Whole lung lysates (for *EGFR* ^L858R^) or tumor nodule lysates (for *EGFR* ^ΔL747–S752^) were probed by immunoblotting using the indicated antibodies. C^+^/L^+^, CCSP-rtTA/tet-o-EGFR^L858R^ bitransgenic mouse; C^+^/Del^+^, CCSP-rtTA/tet-o-EGFR^ΔL747-S752^ bitransgenic mouse; pEGFR Y1092, EGFR phosphorylated at a tyrosine residue at position 1092). (C) Lungs from nontransgenic mice and lacking tumors were treated with placebo or erlotinib in the same manner and analyzed for BIM and EGFR expression status. C^−^/L^−^, nontransgenic mouse. (D) Nude mice bearing PC-9 xenografts were treated with either vehicle control or 25 mg/kg erlotinib intraperitoneally every 12 h × 2 doses. The xenografts were dissected 24 h after the first injection, and tumor lysates were probed by immunoblotting using the indicated antibodies.

We had hoped to extend our studies on the role of BIM in kinase inhibitor-induced apoptosis to human mutant *EGFR* tumor specimens. However, such studies would require precisely timed re-biopsies of tumors in patients just initiating treatment. Since such samples are not currently available, we took advantage of established mutant EGFR mouse lung tumor models. Specifically, we examined the relationship between levels of phospho-EGFR and Bim in tumors induced in transgenic mice that conditionally express mutant L858R EGFRs in lung epithelial cells under the control of tetracycline regulation [9]. We previously showed that treatment with erlotinib eliminated disease within lung tumor-bearing animals [9]. Here, tumor-bearing transgenic animals (as determined by magnetic resonance imaging) were treated with a single dose of vehicle (*n* = 5) or erlotinib (50 mg/kg; *n* = 5) intraperitoneally and humanely killed 13 h later. Lungs from all *EGFR*-transgenic animals expressed the induced oncoprotein, as measured by immunoreactivity of lysates with antisera against the specific L858R EGFR [9] (Figure 7A). At 13 h after treatment, lung tissues from control-treated animals expressed high levels of phosphorylated EGFR, while EGFR phosphorylation was abolished in lungs from erlotinib-treated mice.

We next determined corresponding Bim levels in the mouse lung lysates, using the same BIM antisera as used above to interrogate the human cell lines. Lungs from vehicle-treated L858R mice displayed low levels of Bim EL (Figure 7A). By contrast, corresponding tissue from erlotinib-treated animals displayed elevated levels of Bim. We detected only very low levels of Bim L and did not detect dephosphorylated Bim for unknown reasons (unpublished data). Similar results were obtained by parallel analyses of lung tumor nodules derived from mice expressing the EGFR L747–S752 deletion mutant (*n* = 4) (Figure 7B). By contrast, Bim levels remained constant in non-transgenic mice lacking tumors and treated with either vehicle (*n* = 2) or erlotinib (*n* = 3) in the same manner (Figure 7C).

To verify these results even further using homogenous tissue samples, we treated athymic nude mice bearing PC-9 xenografts with placebo (*n* = 4) or erlotinib (25 mg/kg × 2 doses; *n* = 4) and examined the status of EGFR phosphorylation and BIM in xenograft lysates. As expected, immunoblotting analyses demonstrated that while EGFR was dephosphorylated after erlotinib administration, BIM EL expression was induced (Figure 7D). Conversely, xenograft lysates from placebo-treated animals showed the opposite result—high levels of phospho-EGFR and low levels of BIM EL. Collectively, these studies with both transgenic and xenograft mouse models support our earlier conclusions from in vitro studies on human cells that erlotinib augments the concentration of Bim in lung adenocarcinomas dependent upon mutant EGFR for survival.

### ABT-737 Significantly Enhances Erlotinib-Induced Apoptosis in Drug-Sensitive Lung Adenocarcinoma Cells

ABT-737 is a small-molecule BCL2 antagonist that has antitumor activity, most notably as a single agent against small cell lung cancers, lymphomas, and leukemias [39,40]. ABT-737 also displays synergistic cytotoxicity with chemotherapeutic agents (e.g., paclitaxel) against “less sensitive” cancer cell lines, including ones of non-small cell lung cancer origin (without *EGFR* mutations) [40]. The drug as a single agent has relatively little effect on imatinib-sensitive K562 cells, which harbor the *BCR-ABL* translocation; however, its addition to imatinib greatly enhances imatinib-induced cell death in vitro [23]. Taken together with our findings that mutant EGFR-dependent cells die via the intrinsic pathway of apoptosis, we hypothesized that the intrinsic pathway could be manipulated by ABT-737 to enhance erlotinib-induced cell killing of *EGFR* mutant lung cancer cells.

**Figure 8:**
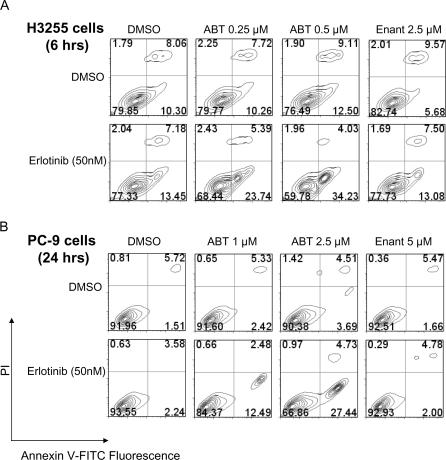
ABT-737 Significantly Enhances Erlotinib-Induced Apoptosis in Drug-Sensitive Lung Adenocarcinoma Cells (A) H3255 cells were cotreated with 50 nM erlotinib and different concentrations of ABT-737 for 6 h. The percentage of apoptotic cells for each drug combination was determined by annexin V/PI apoptosis assays. (B) PC-9 cells were cotreated with 50 nM erlotinib and different concentrations of ABT-737 for 24 h. The percentage of apoptotic cells for each drug combination was determined by annexin V/PI apoptosis assays. The enantiomer of ABT-737 was used a negative control. ABT, ABT-737; Enant, enantiomer.

To verify this hypothesis, we treated H3255 cells for 6 h with different concentrations of ABT-737 alone or in combination with 50 nM erlotinib. Cells were then analyzed for the induction of apoptosis by annexin V/PI assays. Whereas erlotinib or ABT-737 alone induced minimal increases in the percent of apoptotic cells, the combination of the EGFR inhibitor and the BCL2 antagonist significantly increased the percentage of apoptotic cells, in a manner dependent upon the concentration of ABT-737 (Figure 8A). Similar results were obtained with PC-9 cells treated for 24 h (Figure 8B). Cotreatment with erlotinib and the inactive ABT-737 enantiomer, which served as a negative control, had no such effect in either set of cells. These data indicate that BCL2 antagonists such as ABT-737 can be combined with erlotinib to enhance cell death in mutant EGFR-dependent cells.

## Discussion

Selective EGFR kinase inhibitors such as gefitinib and erlotinib induce tumor shrinkage in the majority of patients harboring mutant EGFR lung adenocarcinomas [41]. Studies with cell lines and mouse lung tumor models suggest that such responses involve cell death, but the underlying mechanisms have not been well elucidated. Here, we sought to characterize pathways of apoptosis induced by this class of agents in mutant EGFR-dependent lung adenocarcinoma cells. An enhanced understanding of such processes could lead to the identification of even more effective therapeutic strategies. The studies were motivated by the fact that despite inducing dramatic clinical and radiographic tumor responses, gefitinib and erlotinib rarely induce complete responses and do not cure patients.

Using a panel of human lung cancer cell lines that harbor *EGFR* mutations, we show that mutant EGFR-dependent cells with nanomolar sensitivity to erlotinib undergo G1 cell cycle arrest and cell death in response to kinase inhibition. Cell death is mediated through the intrinsic apoptotic pathway: cells release cytochrome *c* from mitochondria, they display altered localization of the proapoptotic protein BAX, and apoptosis can be blocked by overexpression of the antiapoptotic protein BCL-xL.

We also found that in cells dependent upon mutant EGFR signaling for survival, EGFR kinase inhibition leads to induced levels and dephosphorylation of BIM, a member of the proapoptotic BH3-only family of BCL2-related proteins. Importantly, these changes, which have been shown to enhance the proapoptotic activity of BIM [33,34], are required for erlotinib-induced cell death; knockdown of BIM expression by RNA interference completely eliminates drug-induced cell killing in vitro, and erlotinib dramatically induces BIM levels in cell lines that die but not in cell lines resistant to the drug. Consistent with these in vitro findings, lungs from transgenic mice bearing mutant EGFR-driven lung adenocarcinomas and xenografts of PC-9 cells display increased concentrations of Bim shortly after erlotinib treatment.

BIM has been previously implicated in signaling cell death following cytokine withdrawal [42]. This BH3-only protein has also been shown to be necessary for imatinib-induced killing of BCR-ABL-dependent leukemic cells in vitro (human cells) and in vivo (mice) [23]. The latter study found that only knockdown of Bim and Bad together by RNA interference could completely eliminate imatinib-induced killing. Here we found that knockdown of BIM alone by siRNAs was sufficient to prevent erlotinib-induced cell death in human lung cancer cells. Discrepancies between killing mechanisms in leukemic cells sensitive to imatinib and lung adenocarcinoma cells sensitive to EGFR inhibitors could be explained by different cell types of origin and/or different experimental procedures. Nevertheless, taken together, these findings suggest that the induction of BIM is a common apoptotic mechanism shared by both hematopoietic and epithelial tumor cells that depend upon kinase signaling for survival.

The status of BIM appears to be regulated at multiple points. Transcriptional profiling of erlotinib-treated cells and immunoblotting analysis of cells co-treated with erlotinib and an RNA polymerase II inhibitor indicate that the induction of BIM occurs at least in part at the transcriptional level. Moreover, studies of Bim in NIH 3T3 (mouse) cells transfected with *EGFR* cDNAs and in human lung adenocarcinoma cell lines that harbor *EGFR* mutations indicate that Bim phosphorylation status is influenced by perturbation of EGFR signaling. Use of specific PI3K/AKT and ERK pathway inhibitors on drug-sensitive *EGFR* mutant cells suggests that the ERK pathway predominantly influences BIM status downstream of EGFR. However, others have shown that H3255 and PC-9 cells treated with a more potent and selective MEK1/2 inhibitor, PD0325901 (Pfizer), do not undergo apoptosis (Dr. David Solit, Memorial Sloan-Kettering Cancer Center, New York, personal communication). This finding suggests that (1) while BIM induction is required for erlotinib-induced apoptosis, it may not be sufficient to provoke cell death, and (2) other signaling components downstream of EGFR are also likely to be involved in inducing apoptosis of these cells.

At the clinical level, our results have several implications. Since drug-sensitive *EGFR* mutant cells die via the intrinsic pathway of caspase activation, molecules within this cascade could influence both sensitivity and/or resistance of *EGFR* mutant lung tumor cells to EGFR kinase inhibitors. Conversely, manipulation of this pathway could be a therapeutic strategy to enhance the death of cells that depend upon mutant EGFR signaling for survival. Indeed, the fact that the BCL2 antagonist ABT-737 significantly enhances erlotinib-induced cell killing provides proof-of-principle that such an approach may achieve greater and more durable responses to EGFR inhibitors in patients whose lung tumors harbor *EGFR* kinase domain mutations.

## Supporting Information

Figure S1 Erlotinib Induces Caspase 3/7 Activation in Drug-Sensitive Lung Adenocarcinoma Cell Lines That Harbor *EGFR* Mutations Cell lines were treated with either DMSO or different concentrations of erlotinib in 96-well plates for the indicated times. Caspase 3/7 activity per well was measured as described in the Methods. Values represent the mean ± standard deviation of three independent experiments.
(8.2 MB TIF)

Figure S2 MCL1 Elimination Is Not Required for Erlotinib-Induced Apoptosis in H3255 Cells Cells were treated with erlotinib (100 nM) alone or with erlotinib (100 nM) and the proteasome inhibitor MG132 (10 μM) for the indicated times. Cell lysates were analyzed by immunoblotting with the indicated antibodies.
(8.2 MB TIF)

### Accession Numbers

The GenBank (http://www.ncbi.nlm.nih.gov/) accession numbers for the human genes and proteins discussed in this article are *BAD* (NM_032989), *BAK* (also known as *BAK1*; NM_001188), *BAX* (NM_004324), *BCL-xL* (also known as *BCL2L1*; NM_138578), *BIM* (also known as *BCL2L11*; NM_138621), *EGFR* (NM_005228), *MCL1* (NM_021960).

## Abbreviations

IC_50_: concentration of drug required for 50% growth inhibition
PI: propidium iodide
siRNA: small interfering RNA
TKI: tyrosine kinase inhibitor

## References

[pmed-0040294-b001] Lynch TJ, Bell DW, Sordella R, Gurubhagavatula S, Okimoto RA (2004). Activating mutations in the epidermal growth factor receptor underlying responsiveness of non-small-cell lung cancer to gefitinib. N Engl J Med.

[pmed-0040294-b002] Paez JG, Janne PA, Lee JC, Tracy S, Greulich H (2004). *EGFR* mutations in lung cancer: correlation with clinical response to gefitinib therapy. Science.

[pmed-0040294-b003] Pao W, Miller V, Zakowski M, Doherty J, Politi K (2004). EGF receptor gene mutations are common in lung cancers from “never smokers” and are associated with sensitivity of tumors to gefitinib and erlotinib. Proc Natl Acad Sci USA.

[pmed-0040294-b004] Amann J, Kalyankrishna S, Massion PP, Ohm JE, Girard L (2005). Aberrant epidermal growth factor receptor signaling and enhanced sensitivity to EGFR inhibitors in lung cancer. Cancer Res.

[pmed-0040294-b005] Mukohara T, Engelman JA, Hanna NH, Yeap BY, Kobayashi S (2005). Differential effects of gefitinib and cetuximab on non-small-cell lung cancers bearing epidermal growth factor receptor mutations. J Natl Cancer Inst.

[pmed-0040294-b006] Sordella R, Bell DW, Haber DA, Settleman J (2004). Gefitinib-sensitizing EGFR mutations in lung cancer activate anti-apoptotic pathways. Science.

[pmed-0040294-b007] Tracy S, Mukohara T, Hansen M, Meyerson M, Johnson BE (2004). Gefitinib induces apoptosis in the EGFRL858R non-small-cell lung cancer cell line H3255. Cancer Res.

[pmed-0040294-b008] Ji H, Li D, Chen L, Shimamura T, Kobayashi S (2006). The impact of human EGFR kinase domain mutations on lung tumorigenesis and in vivo sensitivity to EGFR-targeted therapies. Cancer Cell.

[pmed-0040294-b009] Politi K, Zakowski MF, Fan PD, Schonfeld EA, Pao W (2006). Lung adenocarcinomas induced in mice by mutant EGF receptors found in human lung cancers respond to a tyrosine kinase inhibitor or to down-regulation of the receptors. Genes Dev.

[pmed-0040294-b010] Cory S, Huang DC, Adams JM (2003). The Bcl-2 family: roles in cell survival and oncogenesis. Oncogene.

[pmed-0040294-b011] Huang DC, Cory S, Strasser A (1997). Bcl-2, Bcl-XL and adenovirus protein E1B19kD are functionally equivalent in their ability to inhibit cell death. Oncogene.

[pmed-0040294-b012] Vaux DL, Cory S, Adams JM (1988). Bcl-2 gene promotes haemopoietic cell survival and cooperates with c-myc to immortalize pre-B cells. Nature.

[pmed-0040294-b013] Maurer U, Charvet C, Wagman AS, Dejardin E, Green DR (2006). Glycogen synthase kinase-3 regulates mitochondrial outer membrane permeabilization and apoptosis by destabilization of MCL-1. Mol Cell.

[pmed-0040294-b014] Lum JJ, Bauer DE, Kong M, Harris MH, Li C (2005). Growth factor regulation of autophagy and cell survival in the absence of apoptosis. Cell.

[pmed-0040294-b015] Wei M, Zong W, Cheng E, Lindsten T, Panoutsakopoulou V (2001). Proapoptotic BAX and BAK: a requisite gateway to mitochondrial dysfunction and death. Science.

[pmed-0040294-b016] Wolter KG, Hsu YT, Smith CL, Nechushtan A, Xi XG (1997). Movement of Bax from the cytosol to mitochondria during apoptosis. J Cell Biol.

[pmed-0040294-b017] O'Connor L, Strasser A, O'Reilly LA, Hausmann G, Adams JM (1998). Bim: a novel member of the Bcl-2 family that promotes apoptosis. Embo J.

[pmed-0040294-b018] Yu J, Zhang L, Hwang PM, Kinzler KW, Vogelstein B (2001). PUMA induces the rapid apoptosis of colorectal cancer cells. Mol Cell.

[pmed-0040294-b019] Chen L, Willis SN, Wei A, Smith BJ, Fletcher JI (2005). Differential targeting of prosurvival Bcl-2 proteins by their BH3-only ligands allows complementary apoptotic function. Mol Cell.

[pmed-0040294-b020] Willis SN, Adams JM (2005). Life in the balance: how BH3-only proteins induce apoptosis. Curr Opin Cell Biol.

[pmed-0040294-b021] Shinjyo T, Kuribara R, Inukai T, Hosoi H, Kinoshita T (2001). Downregulation of Bim, a proapoptotic relative of Bcl-2, is a pivotal step in cytokine-initiated survival signaling in murine hematopoietic progenitors. Mol Cell Biol.

[pmed-0040294-b022] Ekert PG, Jabbour AM, Manoharan A, Heraud JE, Yu J (2006). Cell death provoked by loss of interleukin-3 signaling is independent of Bad, Bim, and PI3 kinase, but depends in part on Puma. Blood.

[pmed-0040294-b023] Kuroda J, Puthalakath H, Cragg MS, Kelly PN, Bouillet P (2006). Bim and Bad mediate imatinib-induced killing of Bcr/Abl+ leukemic cells, and resistance due to their loss is overcome by a BH3 mimetic. Proc Natl Acad Sci U S A.

[pmed-0040294-b024] Pao W, Miller VA, Politi KA, Riely GJ, Somwar R (2005). Acquired resistance of lung adenocarcinomas to gefitinib or erlotinib is associated with a second mutation in the EGFR kinase domain. PLoS Medicine.

[pmed-0040294-b025] Greulich H, Chen T-H, Feng W, Janne PA, Alvarez JV (2005). Oncogenic transformation by inhibitor-sensitive and -resistant EGFR mutants. PLoS Med.

[pmed-0040294-b026] Shao Y, Gao Z, Marks PA, Jiang X (2004). Apoptotic and autophagic cell death induced by histone deacetylase inhibitors. Proc Natl Acad Sci U S A.

[pmed-0040294-b027] Johnson L, Mercer K, Greenbaum D, Bronson R, Crowley D (2001). Somatic activation of the K-ras oncogene causes early onset lung cancer in mice. Nature.

[pmed-0040294-b028] Janmaat ML, Rodriguez JA, Gallegos-Ruiz M, Kruyt FA, Giaccone G (2006). Enhanced cytotoxicity induced by gefitinib and specific inhibitors of the Ras or phosphatidyl inositol-3 kinase pathways in non-small cell lung cancer cells. Int J Cancer.

[pmed-0040294-b029] Green DR, Kroemer G (2004). The pathophysiology of mitochondrial cell death. Science.

[pmed-0040294-b030] Nishita M, Inoue S, Tsuda M, Tateda C, Miyashita T (1998). Nuclear translocation and increased expression of Bax and disturbance in cell cycle progression without prominent apoptosis induced by hyperthermia. Exp Cell Res.

[pmed-0040294-b031] Salah-eldin A, Inoue S, Tsuda M, Matsuura A (2000). Abnormal intracellular localization of Bax with a normal membrane anchor domain in human lung cancer cell lines. Jpn J Cancer Res.

[pmed-0040294-b032] Nijhawan D, Fang M, Traer E, Zhong Q, Gao W (2003). Elimination of Mcl-1 is required for the initiation of apoptosis following ultraviolet irradiation. Genes Dev.

[pmed-0040294-b033] Luciano F, Jacquel A, Colosetti P, Herrant M, Cagnol S (2003). Phosphorylation of Bim-EL by Erk1/2 on serine 69 promotes its degradation via the proteasome pathway and regulates its proapoptotic function. Oncogene.

[pmed-0040294-b034] Qi XJ, Wildey GM, Howe PH (2006). Evidence that Ser87 of BimEL is phosphorylated by Akt and regulates BimEL apoptotic function. J Biol Chem.

[pmed-0040294-b035] Ley R, Ewings KE, Hadfield K, Cook SJ (2005). Regulatory phosphorylation of Bim: sorting out the ERK from the JNK. Cell Death Differ.

[pmed-0040294-b036] Discafani CM, Carroll ML, Floyd MB, Hollander IJ, Husain Z (1999). Irreversible inhibition of epidermal growth factor receptor tyrosine kinase with *in vivo* activity by *N*-[4-[(3-bromophenyl)amino]-6-quinazolinyl]-2-butynamide (CL-387,785). Biochem Pharmacol.

[pmed-0040294-b037] Ley R, Ewings KE, Hadfield K, Howes E, Balmanno K (2004). Extracellular signal-regulated kinases 1/2 are serum-stimulated “Bim(EL) kinases” that bind to the BH3-only protein Bim(EL) causing its phosphorylation and turnover. J Biol Chem.

[pmed-0040294-b038] Ley R, Balmanno K, Hadfield K, Weston C, Cook SJ (2003). Activation of the ERK1/2 signaling pathway promotes phosphorylation and proteasome-dependent degradation of the BH3-only protein, Bim. J Biol Chem.

[pmed-0040294-b039] Del Gaizo Moore V, Brown JR, Certo M, Love TM, Novina CD (2007). Chronic lymphocytic leukemia requires BCL2 to sequester prodeath BIM, explaining sensitivity to BCL2 antagonist ABT-737. J Clin Invest.

[pmed-0040294-b040] Oltersdorf T, Elmore SW, Shoemaker AR, Armstrong RC, Augeri DJ (2005). An inhibitor of Bcl-2 family proteins induces regression of solid tumours. Nature.

[pmed-0040294-b041] Riely GJ, Politi KA, Miller VA, Pao W (2006). Update on epidermal growth factor receptor mutations in non-small cell lung cancer. Clin Cancer Res.

[pmed-0040294-b042] Villunger A, Scott C, Bouillet P, Strasser A (2003). Essential role for the BH3-only protein Bim but redundant roles for Bax, Bcl-2, and Bcl-w in the control of granulocyte survival. Blood.

